# Plague Victims Catapulted Over Walls Into Besieged City

**DOI:** 10.3201/eid1406.06943

**Published:** 2008-06

**Authors:** Thomas Lux

Early germ
warfare. The dead
hurled this way turn like wheels
in the sky. Look: there goes
Larry the Shoemaker, barefoot, over the wall, and Mary Sausage Stuffer, see how she flies, and the Hatter twins, both at once, soar over the parapet, little Tommy’s elbow bent as if in a salute, and his sister, Mathilde, she follows him, arms outstretched, through the air, just as she did on earth.

